# Chemotherapy for the treatment of alveolar echinococcosis: Where are we?[Fn FN1]

**DOI:** 10.1051/parasite/2024055

**Published:** 2024-09-23

**Authors:** Brice Autier, Florence Robert-Gangneux, Sarah Dion

**Affiliations:** 1 Université de Rennes, CHU Rennes, Inserm, EHESP, Irset (Institut de recherche en santé, environnement et travail) – UMR_S 1085 Rennes France; 2 Université de Rennes, Inserm, EHESP, Irset (Institut de recherche en santé, environnement et travail) – UMR_S 1085 Rennes France

**Keywords:** *Echinococcus multilocularis*, Alveolar echinococcosis, Chemotherapy, Benzimidazole, Nanoparticles

## Abstract

Alveolar echinococcosis (AE) is a severe liver disease due to infection with the *Echinococcus multilocularis* larval stage, called the metacestode. Management of AE is based on benzimidazole chemotherapy (albendazole or mebendazole), associated with surgery when possible. Benzimidazoles are the only compounds recommended for the treatment of AE; however, these are parasitostatic, which means that the parasite can resume growth when treatment is interrupted. Also, benzimidazoles can cause liver dysfunction which may prevent their use. Numerous drugs have been reported to have *in vitro* activity against *E. multilocularis*, but few had satisfactory *in vivo* activity, and none were clearly more effective than benzimidazoles. These drugs belong to various therapeutic categories including anti-infective agents (*e.g.* amphotericin B, mefloquine, pentamidine derivatives), anti-neoplastic compounds (*e.g.* imatinib, nilotinib, bortezomib), plant-extracted compounds (*e.g.* thymol, crocin, carvacrol) and others (*e.g.* metformin, verapamil, thiaclopride). These treatments are generally of limited interest due to their toxicity, their unfavorable pharmacokinetics, or the scarcity of studies involving humans. Apart from benzimidazoles, only amphotericin B, mefloquine and nitazoxanide have been reported to be used for human AE treatment, with unsatisfactory results. Few studies have aimed at developing innovative strategies for AE drug therapy, such as vectorization of drugs using nanoparticles. Altogether, this review emphasizes the urgent need for new therapeutic strategies in AE management, for which there is currently no curative chemotherapy.

## Abstract (fr)

L’échinococcose alvéolaire (EA) est une maladie sévère du foie due à l’infection par la forme larvaire d’*Echinococcus multilocularis*, appelée métacestode. La prise en charge de l’EA repose sur la chimiothérapie par benzimidazolés (albendazole ou mébendazole), si possible associée à la chirurgie. Les benzimidazolés sont les seules molécules recommandées dans le traitement de l’EA, toutefois, ceux-ci sont parasitostatiques, ce qui signifie que le parasite peut reprendre sa croissance lors d’une interruption du traitement. Également, les benzimidazolés peuvent causer une dysfonction hépatique qui peut empêcher leur utilisation. De nombreux médicaments ont été rapportés comme ayant une activité *in vitro* contre *E. multilocularis*, mais peu d’entre eux avaient une activité *in vivo* satisfaisante et aucun n’était clairement plus efficace que les benzimidazolés. Ces médicaments appartiennent à diverses catégories, notamment les agents anti-infectieux (par exemple l’amphotéricine B, la méfloquine, des dérivés de la pentamidine), les composés antinéoplasiques (par exemple l’imatinib, le nilotinib, le bortézomib), les composés extraits de plantes (par exemple le thymol, la crocine, le carvacrol) et d’autres (par exemple metformine, vérapamil, thiaclopride). Ces traitements présentent généralement un intérêt limité en raison de leur toxicité, de leur pharmacocinétique défavorable ou de la rareté des études menées chez l’homme. Outre les benzimidazolés, seules l’amphotéricine B, la méfloquine et la nitazoxanide ont été utilisées dans le traitement de l’EA humaine, avec des résultats insatisfaisants. Peu d’études se sont intéressées à développer des stratégies médicamenteuses innovantes contre l’EA, comme la vectorisation de médicaments à l’aide de nanoparticules. Cette revue souligne le besoin urgent de nouvelles stratégies thérapeutiques dans la prise en charge de l’EA, pour lesquelles il n’existe pas de chimiothérapie curative.

## Introduction

Alveolar echinococcosis (AE) is a liver infection with the larval stage of *Echinococcus multilocularis*, the so-called metacestode [119]. The adult stage of this Taeniid flatworm resides in the small intestine of canids, such as foxes and dogs. This worm lays eggs, which are eliminated in the environment with the feces of the host. In its natural cycle, wild rodents, which are usual intermediate hosts, ingest eggs, which hatch in the intestine and release larvae, which than translocate across the intestinal barrier towards the liver through the portal vein, where they develop further. The larvae consist in an agglomerate of host tissues and parasitic microcysts, each one consisting in a vesicle with a liquid core, an inner layer of cells (germinative layer) and an outer acellular polysaccharidic layer (laminated layer). Humans, as aberrant intermediate hosts, become infected by ingesting eggs through contaminated water, food or hands. The vast majority of infected individuals (99%) rapidly neutralize the parasite and will not develop disease. In the remaining patients, the metacestode will indefinitely grow in the liver, leading to a cancer-like disease leading to death in the absence of treatment [31]. The infection has opportunistic behavior, as immunocompromized individuals are more at risk for developing the disease, with an accelerated course, and a higher risk of metastatic dissemination [7, 76].

The infection is diagnosed through a combination of imaging techniques [ultrasound (US), computed-tomography scanner (CT), magnetic resonance imaging (MRI)] [33], *E. multilocularis*-specific serology (anti-Em18 and anti-Em2+ antibodies detected by ELISA and/or western-blot as a confirmation technique) [51], and/or parasite identification (histopathology or molecular biology such as *E. multilocularis*-specific qPCR or DNA sequencing) [46]. Few techniques are able to assess the viability of the metacestode. Among them, anti-Em18 antibodies are of great value, as their positivity is associated with a viable parasite, but the sensitivity is imperfect as patients with small evolutive lesions can be negative for anti-Em18 [30]. Imaging techniques can be useful for the assessment of parasite viability, especially positron emission tomography (PET) imaging, which can be used to detect the metabolic activity of periparasitic immune infiltrate, which is observed for viable lesions [15]. This technique can lack sensitivity in its standard acquisition (1 h after 18 F-fluorodeoxyglucose [FDG] injection), which can be improved by delaying the acquisition to 3 h after FDG injection and/or by combining PET imaging with CT (PET/CT) or MRI (PET/MRI) [24, 32, 72, 73].

The only curative treatment of AE is surgical excision of the parasite. Imaging does not allow detection of possible small residual parasitic tissues, which can be responsible for relapse. To prevent this, surgery is associated with benzimidazole treatment, such as albendazole (10–15 mg/kg/day) or mebendazole (40–50 mg/kg/day), for at least 2 years [13]. Together with this prophylaxis, clinical and laboratory follow-up is recommended for at least 10 years, but possibly for life depending on comorbidities and the complexity of the disease [87]. If surgery is impossible, the patient will receive palliative treatment which consists of lifelong therapy with one of the benzimidazole drugs mentioned above [13]. As the development or relapse of AE is favored by immunosuppressive therapies, liver transplantation should be strictly limited to patients with severe liver dysfunction, not eligible for partial liver resection, and without metastatic lesions [13, 47]. An increasing number of AE cases are incidentally detected at an early stage of development, thanks to more available and sensitive medical imaging and US screening of at-risk populations [10, 68]. For some of them, treatment with benzimidazole only can be a therapeutic alternative, which can lead to stability or even regression of the lesion [90].

Although AE-related mortality is low in high-income countries [17], management of AE is still limited by the scarcity of therapeutic options. Also, despite the parasite being restricted to the Northern hemisphere, AE is generally considered an emerging infection, especially in Europe and North America [99]. Altogether, this emphasizes the need for a wider range of therapeutic possibilities, from repurposed drugs to innovative strategies, and the development of new compounds. Overall, it is commonly accepted that these drugs must be active on germinative cells, responsible for relapse and metastasis [75]. Given the particular nature of this slowly growing metacestode in the liver tissue, assessing drug efficacy *in vitro* is a difficult task, which is a hindrance to drug development. After an overview of the methods used for drug evaluation, we will review the literature about AE chemotherapy in this article.

## Methods for the assessment of anti-*E. multilocularis* compounds

### *In vitro* screening methods

**Figure 1:**
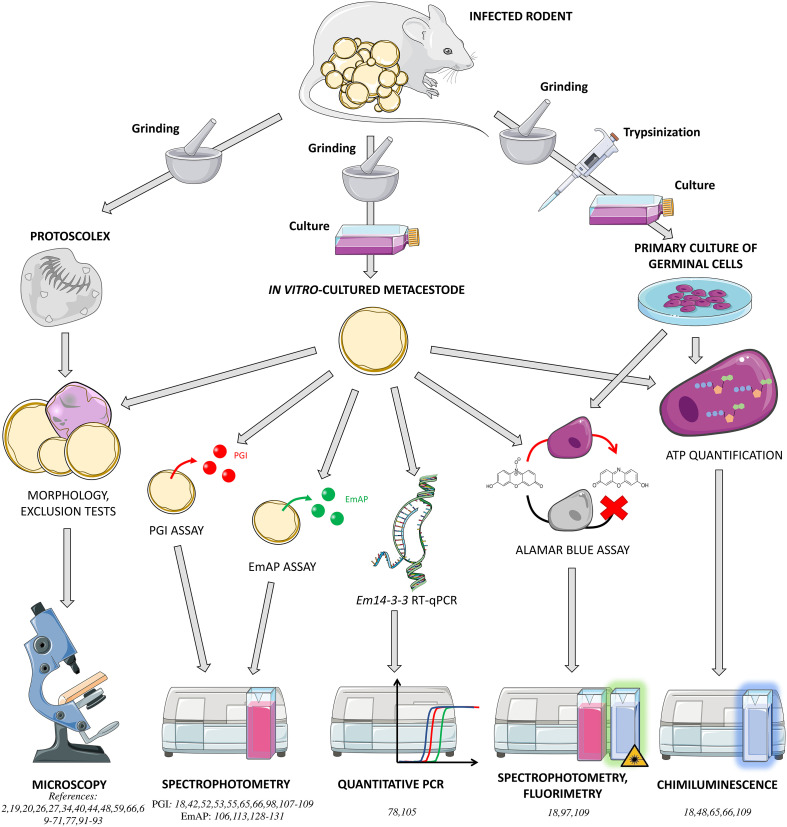
*In vitro* methods for the evaluation of anti-*Echinococcus multilocularis* compounds. *In vitro* methods are based on the exposure of protoscoleces, metacestodes, or cultures of germinal cells to compounds. Different techniques are used for the assessment of drug activity, including morphology and/or exclusion tests, phosphoglucoisomerase (PGI) and the *Echinococcus multilocularis* Alkaline Phosphatase (EmAP) assays, *Em14-3-3* RT-qPCR, the Alamar Blue assay, and ATP quantification. Except for the first one, all of them rely on automated quantification of a signal. However, correlation of these signals with parasite viability remains difficult to predict.

The first *in vitro* evaluations of antiparasitic compounds against *E. multilocularis* were based on protoscoleces, obtained from fertile intermediate hosts [116], or on the *in vitro* culture of metacestodes, leading to the formation of parasitic vesicles [86]. Parasites were then exposed to compounds, and activity was evaluated by the occurrence of morphological alterations, the decrease in the number of vesicles or their survival, assessed by vital staining (*e.g.*, methylene blue or eosin staining) [34, 59] or inoculation to an intermediate host [44]. With the exception of animal inoculation, these methods poorly reflect the parasitocid activity of compounds, as they do not assess their activity on germinative cells. Moreover, they rely on human eye performance, in a qualitative (morphological evaluation) or semi-quantitative (vital staining) manner, with its intrinsic subjectivity (Figure 1).

In order to get rid of these defects, a quantitative *in vitro* assay has been developed in 2010 by the team of Prof Andrew Hemphill (Vetsuiss Faculty, Bern, Switzerland): the phosphoglucoisomerase (PGI) assay. This assay relies on the indirect detection of the enzymatic activity of the EmPGI, an enzyme produced by *E. multilocularis* metacestode and contained in the vesicular fluid and the germinal layer [111]. This enzyme, normally restricted to the inside of the vesicle, is released in case of mechanical lysis or exposure to an active antiparasitic drug [110]. The assay consists in quantifying the EmPGI enzyme in metacestode culture supernatants after 5 to 12 days of exposure to compounds of interest. During the assay, continuous acquisition of absorbance at 340 nm by spectrophotometry allows to determine the kinetics of NADH production, which is proportional to the quantity of EmPGI. The result is expressed as % activity compared to a positive control with 100% activity (*e.g.* Triton X-100). This assay can be used to determine the concentration with 50% activity (EC_50_), and, as adaptable to 24-, 48- or 96-well plates, can be used for screening assays or structure-activity relationship assays [98]. This is currently the most frequently used *in vitro* assay (Figure 1). Of note, this technique does not allow the tester to evaluate the specific activity of compounds against germinative cells, as whole vesicles are used as biological material.

A similar test, based on the detection of an alkaline phosphatase released by *E. multilocularis* (EmAP) [112], has been used by some authors [113, 128–130]. During this assay, the metacestode culture supernatant is added to a buffer solution containing para-nitrophenylphosphate (pNPP), an alkaline phosphatase substrate. After incubation with an active compound, the metacestode releases EmAP in the supernatant, which transforms pNPP into paranitrophenol quantified by absorbance measurement at 405 nm. Drug activity is expressed as the 405 nm absorbance. While this test seems far simpler than the PGI assay, it presents the major drawback of quantifying the final product itself (instead of its kinetics of production), which is not directly related to the quantity of enzyme.

The resazurin assay (Alamar Blue assay) has also been used on metacestodes [109] or on primary cultures of germinative cells [132]. This assay is based on resazurin reduction into resofurin by mitochondria from live cells. The reaction quantification can be spectrophotometric, as both compounds have different absorption spectra, or fluorimetric, as resofurin emits at 590 nm under excitation at 530–560 nm. This technique is poorly sensitive when used on crushed metacestodes, because it does not reflect activity against germinal cells, but against all cell types of the germinative layer. On the other hand, the Alamar assay performed on primary culture of germinative cells poorly reflects the biological reality, as these cells are protected by the outer laminated layer of the metacestode [75]. As an alternative to the Alamar assay, viability of cultured germinative cells can also be assessed by quantification of ATP (CellTiter-Glo^TM^, Promega, Madison, WI, USA).

Two studies evaluated compound activity using an RT-qPCR to quantify the expression of the *Em14-3-3* gene of *E. multilocularis*, by comparison to the actin-coding gene as a housekeeping gene [78, 105]. As the expression of the gene reflects cellular viability, repression of *Em14-3-3* expression following exposure to a compound suggests antiparasitic activity of the compound. However, this method has no longer been used for this purpose since 2008, although no clear reason has been expressed in articles from authors working on *E. multilocularis* [105].

### *In vivo* methods

Activity of compounds against *E. multilocularis* can be evaluated *in vivo*, which encompasses pharmacokinetics and pharmacodynamics variables. Infection can be “primary”, by egg ingestion [114]. Infection can also be “secondary”, *i.e.*, by inoculation of metacestodes or protoscoleces, generally in the peritoneum [114] or less frequently by subcutaneous [42] or intrahepatic inoculation [29] (Table 1). Efficiency of the treatment is usually evaluated through the weight of the lesions, sometimes through their volume or surface. As human infection is mainly hepatic, the best models are those leading to the formation of a liver metacestode, which is closer to human infection in terms of pharmacokinetics. However, these models are constraining, as they require egg isolation or surgical manipulation. Therefore, compounds are more frequently evaluated using models of secondary peritoneal AE.

**Table 1:** Animal experimental models, advantages and drawbacks.

Way of infection	Parasite stage	Similarity to human infection^2^	Source of inoculum	Quantification of inoculum	Handling ease	Example
Gavage	Egg	Way of infection, location	dH	Yes	Medium (Laboratory NSB3)	[134]
Intraperitoneal inoculation^1^	Metacestode	Parasite stage	iH	No	High	[121]
Hepatic inoculation^2^	Metacestode	Parasite stage, location	iH	No	Low (surgery)	[122]
	Protoscolex	Location	iH	Yes	Low (surgery)	[120, 133]

*Note*: dH, definitive host; iH intermediate host.

1 Or subcutaneous.

2 Intraportal, intrahepatic, or subcapsular.

## Active compounds

### Conventional anthelminthic drugs: benzimidazoles

As previously described, management of AE relies on surgical excision of the parasite lesion, considered the only curative method, associated with pre- and post-surgery chemoprophylaxis with a benzimidazole drug, whether albendazole or mebendazole (Table 2) [13]. Activity of benzimidazoles against *E. multilocularis* has been widely reported, using *in vitro* models [40, 44, 93], *in vivo* models [100, 117], or in human cases [21, 127]. Benzimidazoles decreased the infectivity of *in vitro* exposed parasites, and stopped or slowed AE progression when given to infected mammals [54, 100, 117]. Knowledge acquired from their use in human cases also proved their efficacy by the observation of a marked increase in patient survival [21, 127]. It has been shown that patients with severe AE have a life expectancy of more than 10 years if treated, versus less than 2 years without treatment [21, 127]. However, these drugs are only parasitostatic, meaning that if the treatment is discontinued and the parasite is not surgically removed, it can resume its growth [100, 127]. Of note, some reports suggest that long-term therapy with albendazole or mebendazole (more than 2 years of treatment) can be parasitocidal, as it can result in non-viable lesions and/or the absence of recurrence after stopping therapy [4, 5, 9]. Nonetheless, stopping the therapy must involve careful lifelong follow-up to ensure that anti-Em18 antibodies and PET imaging remain negative.

**Table 2:** Practical comparison of albendazole and mebendazole for the treatment of alveolar echinococcosis.

Drug	Albendazole	Mebendazole
Prodrug	Yes	No
Pharmaceutical forms	400 mg tablet	500 mg tablet
	40 mg/mL oral suspension	20 mg/mL oral suspension
Initial dosage	10–15 mg/kg/day	40–50 mg/kg/day
Plasma concentration targets [13]	Peak of albendazole sulfoxide (4 h after administration): 0.65–3 μMol/L	Peak of mebendazole (4 h after administration): >250 nMol/L

Benzimidazoles have a poor biodistribution profile, due to low and/or variable absorption (1–10%, increased when taken with a fatty meal) and rapid metabolization, preventing the use of most of these compounds in human medicine [21, 58, 100]. Importantly, albendazole is a pro-drug of the active albendazole sulfoxide, whereas mebendazole is active itself [22]. Among the factors that interfere with albendazole and mebendazole pharmacokinetics, enzymatic inducers (*e.g.*, ritonavir, rifampicin, phenobarbital, carbamazepine) and inhibitors (*e.g.*, cimetidine) are known to have an impact on their metabolization [101, 118]. These interactions are complex, as they impact the hepatic first-pass effect and metabolization of the drug and its metabolites, resulting in altered half-life and peak concentration. Lifestyle habits are also critical, especially food intake, as this impacts digestive absorption of the drug [81, 126]. Both albendazole sulfoxide and mebendazole have a high distribution volume and are mainly eliminated in the bile as further metabolites.

Benzimidazoles are inhibitors of microtubules polymerization, through binding to tubulin [57]. Their selectivity for parasitic microtubules is related to the amino acid composition of tubulin, especially the 200 residue which is a phenylalanine in susceptible helminths, instead of a tyrosine in mammals and some resistant helminths [95]. Of note, the most expressed *E. multilocularis* tubulin, especially by germinal metacestode cells, is Tub-2, for which the 200 residue is a tyrosine, which could explain the non-parasitocidal activity of benzimidazoles against this parasite [11, 49, 50, 103].

The activity of albendazole could be related to the host immune status. Indeed, it has been described that treatment with albendazole increases periparasite infiltration by immune cells (T CD4^+^ cells, B cells, plasmocytes, macrophages) and decreases the expression of FGL-2, a mediator secreted by T regulator cells (T_regs_) [94]. Moreover, it has been shown that activity of albendazole was lost in athymic mice [123], suggesting that it relies at least partially on the immune system. On the contrary, albendazole treatment of infected mice reduced liver inflammation, as shown by the decrease of tissular IL-1β, IL-6, TNF-α and IFN-γ [124]. Also, in humans, immunocompromized hosts infected with *E. multilocularis* were reported to have a better response to albendazole treatment, with a rapid decrease of lesion size [17]. It should be kept in mind that the immune response in AE is highly complex, and repression of the immune system leads to uncontrolled parasite growth, whereas an excessive Th1/Th2 response, as observed in the late stage of the disease, is responsible for periparasitic fibrosis, limiting diffusion of drugs.

While benzimidazoles are generally well tolerated, the duration of treatment in AE patients (from 2 years to lifelong treatments) increases the risk of adverse drug reactions, such as gastrointestinal disorders, alopecia, myelosuppression (which justifies hematological monitoring), and overall, drug-induced liver injury. To prevent their occurrence and to avoid the use of suboptimal drug dosages, pharmacological monitoring of these drugs is currently recommended, but this is still too rarely available [35]. Therapeutic drug monitoring is performed by determination of plasma levels of albendazole sulfoxide (active metabolite of albendazole) or mebendazole, at peak (4 h after administration) and/or trough (before the following administration) by high-performance liquid chromatography (HPLC) [74, 136]. Liver toxicity is mainly immune-related cytolytic hepatitis; therefore, recurrence is likely in case of benzimidazole reintroduction, preventing its use [82]. Of note, cross-reactivity is not systematic, and intolerance to albendazole may be overcome by switching to mebendazole, and conversely. Also, albendazole is teratogenic if repeatedly administered during the first trimester of pregnancy [16, 25]. This can lead to complex situations of desire for pregnancy during AE treatment, as treatment discontinuation can lead to parasite growth and the hormonal status of pregnant women is suspected to favor metacestode development [6, 8].

Both albendazole and mebendazole are considered well tolerated and highly effective (*i.e.*, at least stabilizing the disease) for the treatment of AE [87]. Albendazole use has become more prevalent over mebendazole, initially because of its lower cost and a simpler drug intake regimen (twice daily for albendazole vs three times daily for mebendazole). Consequently, most studies have focused on albendazole rather than mebendazole (in June 2024: 7,868 results when searching for “albendazole” on PubMed^®^ *vs.* 2,955 results for “mebendazole”), and mebendazole has become less widely available than albendazole. Of note, the cost of both drugs increased in the last decade, but mebendazole was more affected, strengthening its place as second-line therapy [43, 60].

### Salvage treatment: amphotericine B

In case of contraindications to benzimidazoles, the only available alternative is intravenous amphotericin B (AmB), either deoxycholate or liposomal, although its use is not formally recommended [13]. Its use was initially based on the observation of its *in vitro* parasitostatic activity, as shown by the decrease of the number of cultured vesicles. However, new vesicles are produced if the treatment is stopped [92]. In treated patients, AmB led to reduced metabolic activity at PET imaging and stabilization of lesions size [89]. The formulation seems to be important, as a colloidal dispersion of AmB was found to be inactive in a mouse model of peritoneal infection [85]. Also, an interaction was observed with albendazole, as co-exposure to both drugs led to reduced AmB activity *in vitro* [88]. Overall, this treatment is of limited interest in humans as contradictory outcomes have been reported, with stabilization of the disease in some patients, or disease progression in others (2/6 AE patients treated with liposomal AmB between 2008 and 2021 in a case series from the Ulm university hospital, Germany) [14, 115]. However, AmB is currently the only alternative drug therapy to benzimidazoles. Treated patients were empirically administered the lowest dosage (0.5–0.8 mg/kg of deoxycholate AmB or 3 mg/kg of liposomal AmB), with a daily dose for 10–14 days, then once to three times a week, depending on the clinical context and tolerability [14].

### Other compounds

Repurposing of antiparasitic drugs used in other indications is another strategy to develop the arsenal against AE. Among the new candidate drugs for AE chemotherapy, mefloquine stands out by the number of encouraging publications [55, 56, 79, 98, 108]. This compound is already well-known for malaria prophylaxis. It has strong antiparasitic activity *in vitro* against *E. multilocularis*, with an EC_50_ at 30 μM [55, 108]. In animal models of AE, mefloquine was poorly effective when given orally at 25 mg/kg twice a week, but given *via* the intraperitoneal route or at higher doses (100 mg/kg) orally, it led to an effect similar to the current reference treatment, *i.e.*, oral albendazole [55, 56, 98]. Treatment was not parasitocidal, as metacestodes harvested from infected mice were able to reinfect other mice after peritoneal inoculation [56]. The mechanism of action is not yet determined, but could be related to iron metabolism, energy metabolism or cellular transport [79]. To date, only two human AE cases treated with mefloquine have been reported [14, 41]. In both cases, mefloquine was combined with either albendazole or amphotericin B. One patient survived while the other died from secondary cholangitis.

In 2003, strong *in vitro* activity against *E. multilocularis* was reported for nitazoxanide [113], already used in human medicine in other indications, with an *in vitro* EC_50_ of 1.4 μM against metacestodes [54, 91, 110, 113]. However, *in vivo* evaluation of nitazoxanide using the mouse model of secondary peritoneal infection showed lower activity on metacestode weight compared to albendazole [114]. Its effectiveness was also evaluated in a model of primary liver infection, which showed that nitazoxanide-treated mice had significantly fewer lesions compared to the untreated group, without details concerning lesions size or weight [114]. Nitazoxanide has been used for the treatment of patients with contraindications to benzimidazole use and showed negligible activity [14, 45, 115]. Treatment with nitazoxanide led to AE stabilization in only 1/7 reported cases in the literature; the remaining 6 patients had treatment failure or discontinuation of therapy due to side effects (kidney or liver injury). This report marked the end of interest in this drug, at least in the current formulation [14, 35].

Many other compounds have been tested against *E. multilocularis*, with various methods, and studies have yielded variable results. They are summarized in Table 3. These drugs can be gathered into three groups: anti-infectives, anti-neoplastics, or compounds extracted from plants. Repurposing of anti-infective agents seems to be the most suitable strategy, mainly for economic reasons. Of note, antineoplastic agents are known to cause numerous side effects, as they target tumor cells with a high multiplication rate. As *E. multilocularis* germinal cells have a low multiplication rate, as suggested by slow disease progression, antineoplastic agents should be taken with caution, even though some of these compounds have shown *in vitro* activity against *E. multilocularis* metacestodes.

**Table 3:** Other compounds evaluated on *Echinococcus multilocularis* larvae. Effective concentration at 50% (EC_50_) is given for *in vitro* tests when specified in the original publication. Otherwise, the concentration used or the minimum concentration leading to a significant effect is given. For *in vivo* studies, comparison with albendazole is provided, when available.

Compound	*In vitro* method	Animal model	Conclusion	Reference
Anti-infective compounds				
DB1127 (dicationic derivative of pentamidine)	PGI	Peritoneal secondary	Active *in vitro* (EC_50_ = 6.1 μM) and *in vivo* through IP (=ABZ) but not oral route	[52, 108]
Buparvaquone	PGI and resazurin assay	Peritoneal secondary	Active *in vitro* (EC_50_ = 2.9 μM) but not *in vivo*	[97]
Atovaquone	PSC viability	Primary liver	Active *in vitro* (50 μM) and *in vivo* (<ABZ)	[26, 27]
MMV665807	PGI and resazurin assay, PCGC viability	Peritoneal secondary	Active *in vitro* (EC_50_ = 1.2 μM) but not *in vivo*	[109]
Clarithromycin	Metacestode viability	Not applicable	Active *in vitro* (13 μM)	[77]
Artesunate and dihydroartemisinin	EmAP	Peritoneal secondary	Active *in vitro* (40 μM) and *in vivo* (synergy with ABZ)	[106]
Itraconazole	Metacestode viability	Not applicable	Active *in vitro* (1 mM)	[91]
Artemether, caspofungin, miltefosine, rifampicin, cotrimoxazole			Not active *in vitro*	
HIV protease inhibitors	Metacestode and PSC viability	Peritoneal secondary	Nelfinavir active *in vitro* (EC_50_ = 29 μM) and *in vivo* (=ABZ)	[69]
Antineoplastic compounds				
Nilotinib, everolimus	PGI	Subcutaneous secondary	Active *in vitro* (nilotinib EC_50_ = 77 μM, everolimus EC_50_ = 33 μM) but not *in vivo*	[42]
Imatinib	Metacestode morphology, PSC and PCGC viability	Not applicable	Active *in vitro* (25 μM)	[34]
Afatinib, U0126	Metacestode morphology, PSC viability	Peritoneal secondary	Active *in vitro* (10 μM Afatinib, 200 μM U0126) and *in vivo* (=ABZ)	[19]
BI 2536	PCGC viability	Not applicable	Active *in vitro* (10 nM)	[102]
Vincristine, navelbine, methotrexate	Metacestode viability (inoculation)	Not applicable	Vincristine and navelbine active *in vitro* (0.1–60 nM), methotrexate increases parasite growth	[39]
Docetaxel, paclitaxel, doxorubicin, vorinostat, navelbine	Metacestode viability (inoculation)	Not applicable	Docetaxel (2–10 μM) and paclitaxel (2–10 μM) active *in vitro*	[38]
Bortezomib	PGI	Peritoneal secondary	Active *in vitro* (EC_50_ = 0.6 μM) but not *in vivo*	[107]
PIM kinases inhibitors: SGI-1776, CX-6258	Metacestode morphology, PCGC viability	Not applicable	Active *in vitro* (10 μM)	[48]
2-methoxyestradiol	*Em14-3-3* RT-qPCR	Peritoneal secondary	Active *in vitro* (5 μM) but not *in vivo*	[105]
2-deoxy-D-glucose	EmAP	Peritoneal secondary	Active *in vitro* (80 μM) and *in vivo* (=ABZ)	[128]
3-bromopyruvate	EmAP	Peritoneal secondary	Active *in vitro* (40 μM) and *in vivo* (=ABZ)	[130]
Lonidamine	EmAP	Not applicable	Active *in vitro* (40 μM)	[131]
Plant-extracted compounds				
Osthole	Not applicable	Peritoneal secondary	Active *in vivo* (=ABZ)	[135]
Ampelopsin	EmAP	Not applicable	Active *in vitro* (40 μM)	[129]
Thymol	PSC viability	Peritoneal secondary	Active *in vitro* (67 μM) and *in vivo* (=ABZ)	[2, 3]
Carvacrol	Metacestode viability	Peritoneal secondary	Potentialization of ABZ *in vitro* and *in vivo*	[71]
Crocin	PGI, PSC and PCGC viability	Peritoneal secondary	Active *in vitro* (EC_50_ = 11.4 μM) and *in vivo* (=ABZ)	[65]
Others				
Metformin	PSC and PCGC viability	Peritoneal secondary	Active *in vitro* (10 mM) and *in vivo*	[70]
Endochin-like quinolones	PGI and resazurin assay, PCGC viability	Not applicable	Active *in vitro* (EC_50_ = 0.2–1.7 μM)	[18]
Verapamil	Not applicable	Liver secondary	Active *in vivo* (=ABZ)	[29]
Thiacloprid	PGI and PSC viability	Peritoneal secondary	Active *in vitro* (5 μM) et *in vivo* (=ABZ)	[66]
Isoprinosine, inosine, L-Phe-Phe-Ome	PSC viability	Not applicable	Active *in vitro* (inosine and L-Phe-Phe-Ome EC_50_ ≈ 50 μM, isoprinosine EC_50_ ≈ 250 μM)	[59]
R-propylamine	PSC viability	Peritoneal secondary	Active *in vitro* (20 μM) and *in vivo* (=ABZ)	[20]
Ruthenium complexes	PGI	Not applicable	Active *in vitro* (EC_50_ = 1.4–4.7 μM)	[53]

*Note*: ABZ, albendazole; PCGC, primary culture of germinative cells; EmAP, *E. multilocularis* alkaline phosphatase assay; IP, intraperitoneal; PGI, phosphoglucose isomerase assay; PSC, protoscolex; =ABZ, equivalent to albendazole; >ABZ, superior to albendazole; <ABZ, inferior to albendazole.

## New therapeutic strategies

During the two last decades, nanoparticle-based vectorization has been recognized as a major innovation in drug administration [80]. These new formulations rely on the synthesis of 1–1,000 nm compound-containing particles, with the aim of modifying the pharmacokinetic properties. Depending on the type of nanoparticle, the drug can be linked to its surface, encapsulated into a hydrophilic or lipophilic core, or loaded into the matrix composing the nanoparticle. Three types of nanoparticles are classically described, *i.e.*, lipid-based, polymeric, and inorganic nanoparticles [12]. Few nanoparticular therapies have been validated to date for administration to humans [83]. They are mostly used for drug vectorization (mainly antineoplastic agents); some inorganic formulations are indicated for iron supplementation, medical imaging, or photothermal therapy. Other usages are under development and evaluation, such as genetic therapy or immunotherapy [80]. Nanoparticle properties generally depend on their composition (Table 4), but many chemical methods have been developed to optimize them, for example by modifying their surface, their size, or their shape. It is also possible to address nanoparticles to targeted cells, which improves their efficacy and decreases the occurrence of side effects. With this aim, cell receptors or specific antibodies can be grafted onto the surface of nanoparticles.

**Table 4:** Main characteristics of therapeutic nanoparticles.

	Examples	Tolerance	Stability	Cost	FDA-approved use
Lipid-based	Liposomes	+++	+/−	+/++	Drug vectorization
	Nanocapsules				
	Solid nanocarriers				
	Emulsions				
Macromolecular	Polymeric micelles	++	++	+/++	Drug vectorization
	Polymersomes				
	Nanospheres				
	Dendrimeres				
Inorganic	Gold	+/−	+++	+++	Drug vectorization
	Iron				Iron supplementation
	Silica				Imaging

*Note*: Inspired from [12, 80, 83]. Semi-quantitative evaluation: − poor, + low, ++ medium, +++ high.

Most new formulations for AE chemotherapy rely on nanoparticular vectorization of drugs [1, 23, 61, 63, 64, 96, 125]. The first report was published in 1993 and showed the efficacy of doxorubicin coupled with polyisohexylcyanoacrylate nanoparticles, in a mouse model of secondary liver infection [64]. The treatment did not modify the size of the lesions, but decreased their viability, as shown by the lower proportion of infected gerbils, *Meriones unguiculatus*, after inoculation of the metacestodes. In the 1990s, a study evaluated the activity of albendazole-loaded poly-D,L-lactide nanoparticles, in a mouse model of secondary liver infection [96]. The formulation was effective at low doses, but not at high doses, putatively due to toxicity on macrophages. Four studies evaluated the efficacy of liposomal albendazole, including 3 experimental studies which showed that the liposomal formulation was at least as effective as tablets of albendazole in secondary peritoneal models of infection [1, 23, 125]. The remaining study reported a series of 12 AE patients treated with liposomal albendazole in the Xinjiang district of China [61]. The authors concluded on effectiveness of the therapy in 75% of patients (9/12), and a lack of activity in 25% of patients (3/12), based on PET/CT imaging. However, no comparison to a control group treated with tablets of albendazole was carried out. Another formulation of albendazole in chitosan nanoparticles, a chitin-like polyoside, showed higher efficacy than tablets of albendazole in a mouse model [1]. Recent studies evaluated the vectorization of new drugs so-called E2-a (extracted from the Fabaceae *Sophora moorcroftiana*) and H1402 (carbazole aminoalcohol) using poly-lactic co-glycolic acid (PLGA) nanoparticles [62, 63]. The formulations were as effective as oral albendazole for the treatment of secondary infections (peritoneal or hepatic AE).

Other therapeutic strategies are rare and only consist of miscellaneous formulations of benzimidazoles, such as solid dispersions [28, 37, 84], and new tablet formulations containing hydroxypropyl methylcellulose [36], emulsion [104], or oily suspensions [67] for use *via* the oral route.

## Conclusions

Benzimidazoles are the only recommended drugs for AE treatment. However, they are parasitostatic, meaning that the parasite can continue to grow in case of discontinuation. Additionally, benzimidazoles may be responsible for liver dysfunction that can prevent their use. Amphotericin B can be used as rescue treatment, but it can have severe side effects and its efficacy appears to be variable. Finally, numerous drugs have been reported to have some *in vitro* activity against *E. multilocularis*, but few had satisfactory activity *in vivo*, and none were clearly more effective than benzimidazoles in humans. There is an urgent need for new therapeutic strategies for the management of AE, a disease for which there is no curative chemotherapy.
